# Wireless Biosensor System for Real-Time l-Lactic Acid Monitoring in Fish

**DOI:** 10.3390/s120506269

**Published:** 2012-05-11

**Authors:** Kyoko Hibi, Kengo Hatanaka, Mai Takase, Huifeng Ren, Hideaki Endo

**Affiliations:** Department of Ocean Science, Tokyo University of Marine Science & Technology, Konan 4-5-7 Minato-ku, Tokyo 108-8477, Japan; E-Mails: hibi@kaiyodai.ac.jp (K.H.); ken5hatanaka@gmail.com (K.H.); danzhime.anden.see@gmail.com (M.T.); hf-ren@kaiyodai.ac.jp (H.R.)

**Keywords:** l-lactic acid, fish, wireless, monitoring, interstitial fluid

## Abstract

We have developed a wireless biosensor system to continuously monitor l-lactic acid concentrations in fish. The blood l-lactic acid level of fish is a barometer of stress. The biosensor comprised Pt-Ir wire (φ0.178 mm) as the working electrode and Ag/AgCl paste as the reference electrode. Lactate oxidase was immobilized on the working electrode using glutaraldehyde. The sensor calibration was linear and good correlated with l-lactic acid levels (R = 0.9959) in the range of 0.04 to 6.0 mg·dL^−1^. We used the eyeball interstitial sclera fluid (EISF) as the site of sensor implantation. The blood l-lactic acid levels correlated closely with the EISF l-lactic acid levels in the range of 3 to 13 mg·dL^−1^ (R = 0.8173, n = 26). Wireless monitoring of l-lactic acid was performed using the sensor system in free-swimming fish in an aquarium. The sensor response was stable for over 60 h. Thus, our biosensor provided a rapid and convenient method for real-time monitoring of l-lactic acid levels in fish.

## 1. Introduction

Due to the rapidly increasing human population, there is a growing fear of the possible depletion of food resources. Marine products are an important food resource in countries surrounded by the sea, such as Japan. The fish catch is declining due to fishing restrictions imposed due to overexploitation and marine pollution, thus fish cultivation is beginning to attract attention in the marine products industry. The fish cultivation industry has various problems to overcome, such as food safety issues. Antibiotics are routinely used to prevent fish disease, and human consumption of the residual antibiotics remaining in the food is a major concern. To increase fish farm efficiency, large quantities of fish are often bred within a limited area. The overcrowded breeding environment leads to a rapid decrease in the water quality and exposure of the cultivated fish to excessive stress, which reduces the resistance of the fish to disease.

In an overcrowded breeding environment, a single sick individual can quickly spread disease throughout the entire fish culture. Such disease outbreaks can produce enormous economic damage. As antibiotics are used to treat sick fish, drugs may remain in the body of the treated fish, which leads to widespread concern that residual antibiotics will have negative effects on consumer health.

Maita *et al.* reported that the health of cultivated fish can be effectively monitored using a blood test to measure blood components such as glucose, cholesterol, and l-lactic acid [1,2]. Stress, such as transport stress, pesticide exposure, and oxygen deficiency, increases blood l-lactic acid levels [3–5]. Kamalaveni *et al.* reported that blood l-lactic acid levels increase in fish exposed to nerve poison [3]. Ramikrishna *et al.* also reported increases in blood l-lactic acid levels in carp exposed to sublethal concentrations of waste residues from distillation processes [4].

In addition, Hur *et al.* reported that transportation stress increases l-lactic acid levels in the blood of flatfish [5]. l-Lactic acid, which is the final product of sugar metabolism and the glycolytic pathway, is caused by the reduction of pyruvic acid by the catalysis of lactic acid dehydrogenase. Lactic acid is produced from muscle cells under anaerobic conditions, and is then converted to energy after being used for glucose-resynthesis in the liver [6]. Because both blood glucose and l-lactic acid levels are increased by excessive stress, blood levels of these compounds are good indicators of stress in fish. Therefore, studies on the management of fish health by inspecting the blood constituents revealed that l-lactic acid levels can be used as an indicator of stress levels in fish [3–5]. Measuring blood l-lactic acid levels, however, requires difficult procedures such as preprocessing blood to obtain blood plasma, which decreases the practical applicability of this process.

We reported the development of a needle-type biosensor for monitoring blood glucose in fish in 2006 [7]. Using this technique, long-term measurements were difficult to achieve because the sensor output current was decreased by blood coagulation and protein (e.g., albumin, γ-globulin) coalescing on the sensor. Methods for continuous *in vivo* monitoring of glucose concentrations in humans and other mammals have been studied, focusing on the measurement of glucose in interstitial fluid (ISF) [8–10]. The ISF in fish is found behind the eyeball and can be accessed by gently pushing the eyeball to the side near the snout. We confirmed that glucose levels in the ISF are highly correlated with those in blood based on 112 samples of fish eyeball ISF (y = 2.2996 + 0.9438x, R = 0.960) [8]. We named this ISF ‘eyeball interstitial sclera fluid’ (EISF). We then developed a fish body-implantable needle-type glucose biosensor connected to a wireless monitoring system [9]. Implantation of the biosensor in the EISF allowed for real-time monitoring of blood glucose levels in fish. The needle-type glucose biosensor we developed is a dipole biosensor comprising a platinum iridium wire as the working electrode and Ag/AgCl as the counter electrode [8–11]. To limit the effect of impurities in the EISF, a 5% Nafion solution was applied to the working electrode and glucose oxidase (GOx) was immobilized onto the electrode by forming an enzyme layer on top of the Nafion layer. The sensor output current in glucose standard solutions had a very strong correlation (R = 0.9983) with glucose concentrations within the range of 0.18 to 144 mg·dL^−1^ [9]. The blood glucose level of the fresh water fish (tilapia) is reported to be ∼50 to 100 mg·dL^−1^ [12]. Therefore, this sensor is applicable for measuring glucose levels in fish. By connecting the sensor to a wireless, radio wave-based monitoring system and, we were able to continuously measure blood glucose levels for 72 h [9].

In 1996, Yoon *et al.* developed a stick-type biosensor for measuring l-lactic acid concentrations [13]. A method for measuring blood l-lactic acid concentrations in fish, however, has not been reported to date.

Based on our understanding of fish stress responses during breeding, real-time monitoring of blood l-lactic acid concentration and blood glucose levels in fish will contribute to maintaining disease-free fish aquaculture. The purpose of this study was to develop a method for real-time monitoring of l-lactic acid concentrations in the fish blood using a biosensor. We first investigated the relationship between EISF and blood l-lactate acid levels in fish. We then developed a needle-type enzyme sensor, evaluated the performance of this sensor, and examined the possibility of measuring l-lactic acid concentrations in fish blood using a wireless monitoring method.

## 2. Experimental Section

### 2.1. Reagents

Lactic acid oxidase (LOX) from *Pediococcus* sp.; E.C. 1.13.12.4, 100 U/g was purchased from Sigma-Aldrich (St. Louis, MO, USA). Bovine serum albumin (BSA), 5% Nafion dispersion solution, 2-phenoxy ethanol, and heparin sodium (10,000 U/g) were purchased from Wako Pure Chemical Industries (Tokyo, Japan). Glutaraldehyde solution (25%) was purchased Tokyo Chemical Industries (Tokyo, Japan). A lactic acid standard solution was prepared by dissolving lactic acid in 0.1 M phosphate-buffered saline (PBS).

### 2.2. l-Lactic Acid Analysis to Evaluate the Relationship between l-Lactic Acid Concentration in Blood and EISF

We used Nile tilapia (*Oreochromis niloticus*), cultured at the Tokyo University of Marine Science & Technology, as the test fish. Each fish (body weight; *ca.* 150–300 g, body length; 15–25 cm) was netted from the preserve and anesthetized with 400 ppm 2-phenoxyethanol by bath exposure for 5 min. Blood samples were collected from the caudal vein along the backbone by inserting a heparinized syringe fitted with a 23G needle (0.65 mm × 25 mm). The blood samples (50–100 μL) were then centrifuged (550 g) for 10 min to separate the plasma. EISF samples were collected by inserting a syringe fitted with a 27G needle (0.40 mm × 19 mm) into the sclera of the eyeball. The collection procedure for each fish was completed within 5 min to minimize stress-induced measurement errors. Each collected sample was transferred to a test tube and kept at −80 °C until analysis.

l-Lactic acid concentrations were determined using an enzymatic colorimetric method (EnzyChrom l-Lactate Assay Kit, BioAssay Systems, Hayward, CA, USA) as the conventional method. Each sample (20 μL) was mixed with 80 μL assay buffer containing enzyme (lactate dehydrogenase) and a color-producing reagent. Absorbance at 550 nm was measured at 0 and 20 min after starting incubation at room temperature. The lactate assay kit is based on the lactate dehydrogenase-catalyzed oxidation of lactic acid, in which NADH formed by the reaction reduces a formazan reagent. The intensity of the product color, measured at 550 nm, is proportionate to the lactic acid concentration in the sample.

### 2.3. Temporal Change in the Lactic Acid Concentration in Blood and EISF in Individual Fish over Three Weeks

We evaluated whether the temporal change in l-lactate concentrations in the blood was reflected by changes in the l-lactic acid concentration in EISF in individual fish. The testing system comprised a 56 L plastic tank (60 × 30 × 36). During the test period, the water temperature was maintained at 30 °C with continuous aeration and recirculation through a biologic filter. Blood and EISF sample were collected between 10:00 and 14:00 daily. We fed the fish every day after collecting the blood samples.

### 2.4. Preparation of the Needle-Type Biosensor

**Figure 1.**
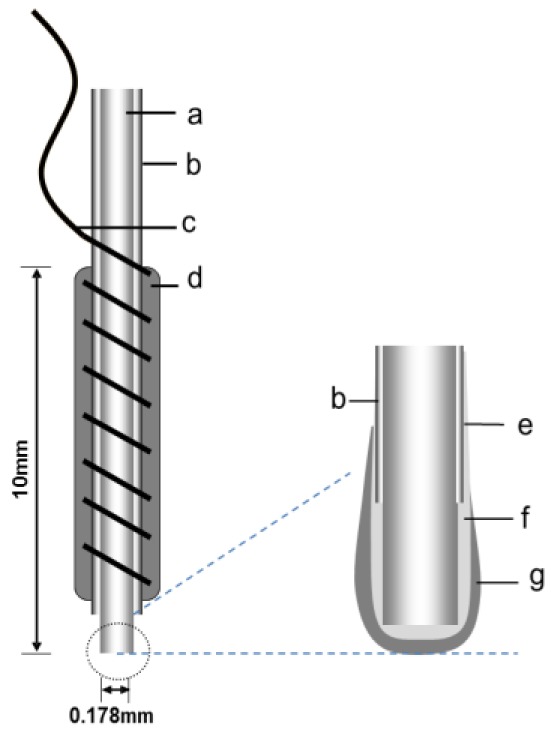
Schematic diagram of the needle-type biosensor. (**a**) Pt-Ir wire, (**b**) Teflon layer, (**c**) Cu wire, (**d**) Ag/AgCl paste, (**e**) Nafion layer, (**f**) Working electrode, (**g**) LOX layer.

Figure 1 shows the structure of the needle-type biosensor. The system comprised a Pt-Ir wire working electrode and an Ag/AgCl reference/counter electrode. As a working electrode, 15-mm Pt-Ir cylinders with a 178-μm radius were prepared by cutting strips of Teflon-coated wire. A sensing cavity was prepared by stripping the Teflon along the wire to expose 2.0 mm of metal. Copper wire was wrapped around the Teflon-coated surface as lead wire. To use the Ag/AgCl paste as a reference electrode/counter electrode, it was applied to the Teflon wrapped around the copper wire. The tip of the wire was sealed with epoxy resin, leaving a 0.7 mm-long sensing cavity. The working electrode coated in a Nafion dispersion solution and air dried for 30 min, and this procedure was repeated. The working electrode was then coated with the enzyme solution using a microsyringe, and then stored at 4 °C for 30 min. The enzyme solution was prepared by dissolving 2.5 mg BSA and 1.17 mg LOX to 0.1 M PBS (pH 7.0), and was kept at −80 °C until use. The LOX was immobilized by dipping the enzyme-coated electrode in glutaraldehyde (1%) for 2 s, and then stored at 4 °C for 30 min. The glutaraldehdye cross-linked the LOX to the BSA. After washing with PBS, the needle-type biosensor was stored in PBS (pH 7.8) overnight at 4 °C.

### 2.5. Amperometric l-Lactic Acid Measurement

The enzyme sensor was connected to a multichannel potentiostat (3100, Pinnacle Technology Inc, Lawrence, KS, USA) attached to a personal computer (DELL Dimension 4700C, Round Rock, TX, USA) via either a USB 2.0 or RS232C port. A constant potential was maintained using the potentiostat. A 650 mV potential (*vs.* Ag/AgCl) was applied to the Pt-Ir working electrode using the potentiostat. Amperometric l-lactic acid measurement was then made from the surface of the working electrode based on the following equations:

(1) $$\text{L-Lactic acid}+{\text{O}}_{2}---(\text{LOX})\to \text{Pyruvate}+H_{2}O_{2}$$

(2) $$H_{2}O_{2}---(\text{electrocatalytic oxidation})\to O_{2}+2H^{+}+2e^{-}$$

Reaction (2) produces an electric output current that is recorded via the computer-controlled data acquisition software PAL (Pinnacle Acquisition Laboratory). The sensor was placed in 50 mL of 0.1 M PBS (pH 8.0). After the output current stabilized, a 2 μL aliquot of the 1,000 mg·dL^−1^ lactic acid standard solution was added. This procedure was repeated until 25 aliquots had been added. The sensor output current was measured after each addition of l-lactic acid and the background current was subtracted from the response current.

### 2.6. l-Lactic Acid Monitoring in Fish

#### 2.6.1. Sensor Insertion and Fixation

Sensor implantation and fixation were performed after anesthetizing the fish with 400 ppm 2-phenoxyethanol. A small hole was created in the sclera using a 20G catheter (φ1.10 mm) comprising an outer Teflon layer and inner puncture needle (20G, Serflow^TM^ Terumo, Tokyo, Japan). The inner puncture needle was then removed, and the excess exposed catheter on the skin was trimmed off. A sensor was then inserted into the scleral ISF, immersing the working and reference electrodes of the sensor in the EISF. The sensor was then fixed in place using a biomedical adhesive (Aronalpha-A Sankyo, Toagosei, Tokyo, Japan). The adhesive was applied around the insertion point to fill the hole and the sensor was stapled to the fish's body. After implantation and fixation, the sensor was attached to a multichannel potentiostat. Monitoring of l-lactic acid was performed after the background current of the sensor stabilized.

#### 2.6.2. *In Vivo* Calibration

Continuous estimation of l-lactic acid in blood was performed using both one-point and two-point *in vivo* calibration methods [14–16]. For the one-point calibration method, sensor sensitivity (*S*) was determined from a single blood l-lactic acid measurement as the ratio between the l-lactic acid in the blood (*G*) and the sensor output current (*I*). l-Lactic acid levels were then estimated from the current (*I*) using Equation (3):

(3) $$G_{\left(t\right)}=I_{\left(t\right)}/S$$

The sensor output current was first allowed to stabilize after the sensor was implanted into the sclera. l-Lactic acid in blood samples was then obtained from the caudal vein, and the l-lactic acid in blood level (*G*) was measured. These parameters (*G* and *I*) were used as the calibration reference. For the two-point calibration method, the sensor sensitivity [*S*; expressed in nA/(mg dL^−1^)] was determined as well as the sensor output current intercept (*I*_0_; expressed in nA), *i.e.*, the theoretical sensor output that would be observed in the absence of l-lactic acid in ISF as *I*_0_ = *I*_1_ − *G*_1_ × S. These parameters, *S* and *I*_0_, were subsequently used to transform the sensor output *I*(*t*) to estimate l-lactic acid levels *G*(*t*) using Equation (4):

(2) $$G(t)=(I(t)-I_{0})/S$$

The method of determining S and I_0_ takes into account the change in the sensor current (*I*) from *I*_1_ to*I*_2_, namely, a *S* = (*I*_2_ − *I*_1_)/(*G*_2_ − *G*_1_) and *I*_0_ = *I*_1_ − (*S* × *G*_1_) during a change in l-lactic acid in blood levels “*G*” from “*G*_1_” to “*G*_2_”.

#### 2.6.3. Wireless System for Monitoring l-Lactic Acid under Free-Swimming Conditions

**Figure 2.**
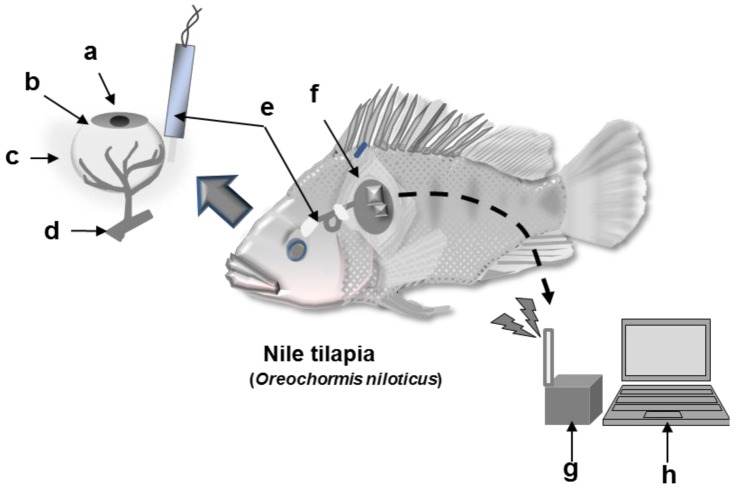
Schematic diagram of the wireless monitoring system for fish. (**a**) Eyeball, (**b**) Sclera, (**c**) EISF, (**d**) Blood vessel, (**e**) Needle-type l-lactic acid sensor, (**f**) Wireless potentiostat, (**g**) Receiver, (**h**) Personal computer.

A schematic diagram of the wireless monitoring system is shown in Figure 2. Sensor implantation and fixation were performed according to the same method used for the wired monitoring. The sensor was connected to a wireless potentiosat (3102BP, Pinnacle Technology Inc.) and covered with a waterproof polypropylene sheet. The waterproofed wireless potentiostat was attached to the dorsal and pectoral fins of the fish using nylon thread. The signal of the sensor output current signal was sent to a receiver (3100RX, Pinnacle Technology Inc) using a radio wavelength of 916.5 MHz. The fish were then transferred to a water tank (40 cm × 24 cm × 50 cm). During the monitoring, EISF samples from the contralateral eyeball were collected periodically from fish anesthetized with 400 ppm 2-phenoxy-ethanol to prevent noise signals due to fish movement. After collecting the EISF, the fish was placed in a water tank that did not contain 2-phenoxyethanol. During the experiment, the water was maintained at 30 °C, aerated, and filtered. Continuous monitoring of l-lactic acid was performed after the sensor output current stabilized.

## 3. Results and Discussion

### 3.1. Comparison of l-Lactic Acid between Blood and EISF

**Figure 3.**
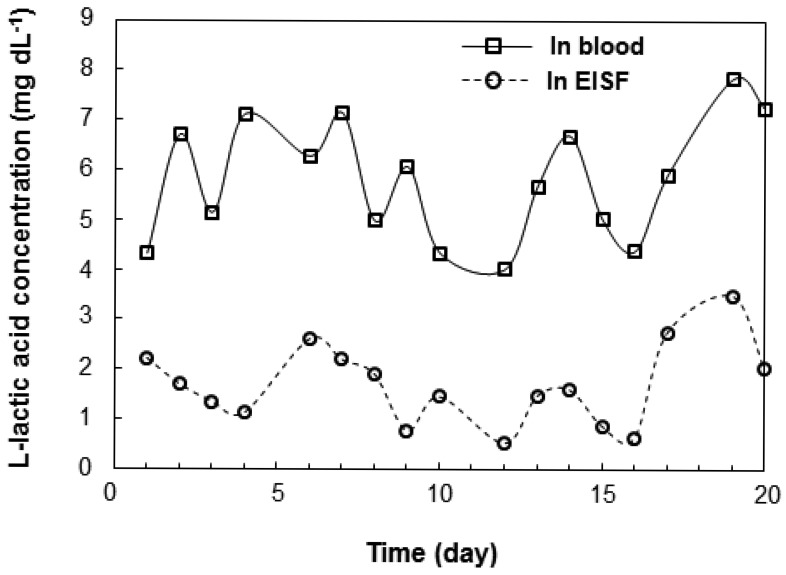
Representative plot of l-lactic acid concentration in the blood and EISF in an individual fish l-Lactic acid concentrations in blood are plotted as white circles and those in EISF are plotted as black circles.

**Figure 4.**
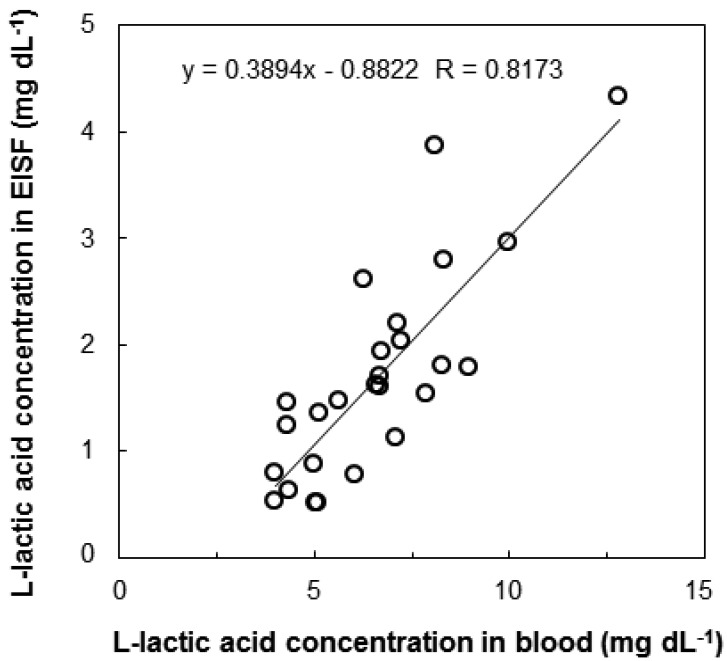
Correlation of l-lactic acid concentration in blood and EISF in randomly selected fish.

We investigated the correlation between l-lactic acid concentrations in the blood and EISF. Changes in the l-lactic acid concentration of the blood and EISF were measured over time in individual fish. The changes in the l-lactic acid concentration in the blood and EISF were similar (Figure 3). After l-lactic acid is produced in the ground substance of the muscles, it diffuses outside the cells, and travels through the blood to the ISF. Therefore, there is somewhat of a time-lag between the concentration of l-lactic acid in the blood and the EISF. We then examined the interindividual variations in the relationship between blood and EISF l-lactic acid concentrations. Fish (n = 26) were captured randomly from the aquarium, and both blood and EISF samples were obtained. The correlation between blood and EISF l-lactic acid levels in the range of 3 to 13 mg·dL^−1^ was very strong (R = 0.8173, Figure 4). The l-lactic acid concentration in EISF was approximately 30% that of the l-lactic acid concentration in blood. Based on these findings, the blood l-lactic acid concentration can be reliably estimated by measuring the EISF l-lactic acid concentration. The area around the eyeball containing ISF is useful as a sensor insertion site for estimating the l-lactic acid concentration in blood.

### 3.2. Characteristics of the Needle-Type l-Lactic Acid Biosensor

**Figure 5.**
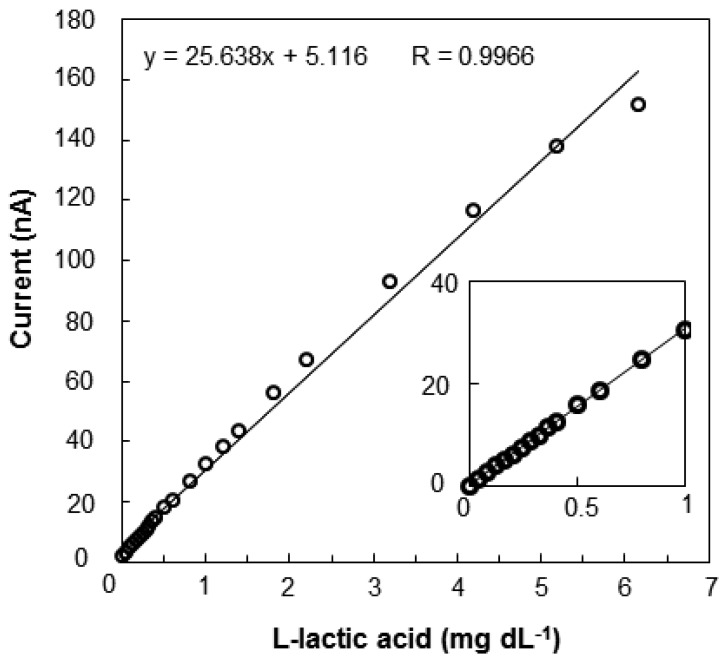
Relationship between sensor output current and L-lactic acid concentration. The measurement was performed by sequential addition 2 μL of 1,000 mg·dL^−L^ L-lactic acid standard solution in PBS (pH 8.0, 30 °C) under a +650 mV potential (*vs.* Ag/AgCl).

**Figure 6.**
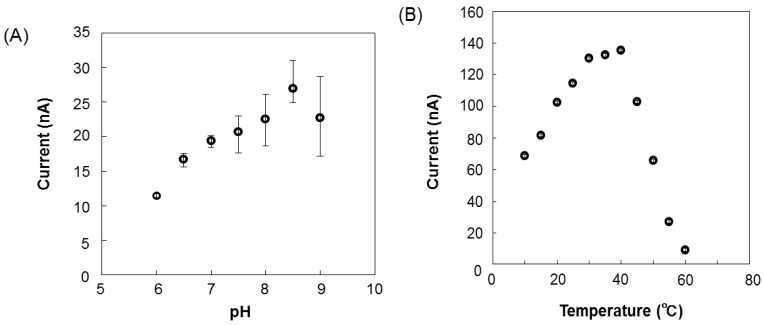
Typical effects of pH (**A**) and temperature (**B**) on the l-lactic acid sensor response. In (A) the temperature was 30 °C, in (B) the pH was 8.0. The measurements were performed using 5 mg·dL^−1^ l-lactic acid solution under a +650 mV potential (*vs.* Ag/AgCl).

The relationship between the output current and l-lactic acid concentration is shown in Figure 5. The calibration curve of the l-lactic acid concentration was linear from 0.04 to 6.0 mg·dL^−1^ (y = 6.0354x − 9.428, R = 0.9944), and the detection limit was 0.04 mg·dL^−1^. After the baseline stabilized, an aliquot of a standard l-lactic acid solution was added. The l-lactic acid concentration could be rapidly measured, because even the response required only 2 min to stabilize. As the sensor comprises a biologic catalyst (enzyme), the response can vary depending on the pH and temperature of the solution in which the immobilized enzyme is placed. The effects of pH and temperature on the sensor induced current for a 3 mg·dL^−1^ standard solution are shown in Figure 6. The sensor current gradually increased as the pH increased from 6.0 to 8.5 and then rapidly decreased at pH greater than 8.5 (Figure 6(A)). The pH of EISF in Nile tilapia is 8.0 [8]. Although pH affects enzyme activity, even at pH 8.0 the sensor induced enough current to detect changes in the l-lactic acid concentration. Temperature also has important effects on enzyme activity. For temperatures ranging from 10 to 60 °C, the sensor current increased rapidly up to 40 °C and rapidly decreased at temperatures above 40 °C (Figure 6(B)). Enzyme activity of the electrode surface decreased with a temperature increase to above 40 °C.

**Figure 7.**
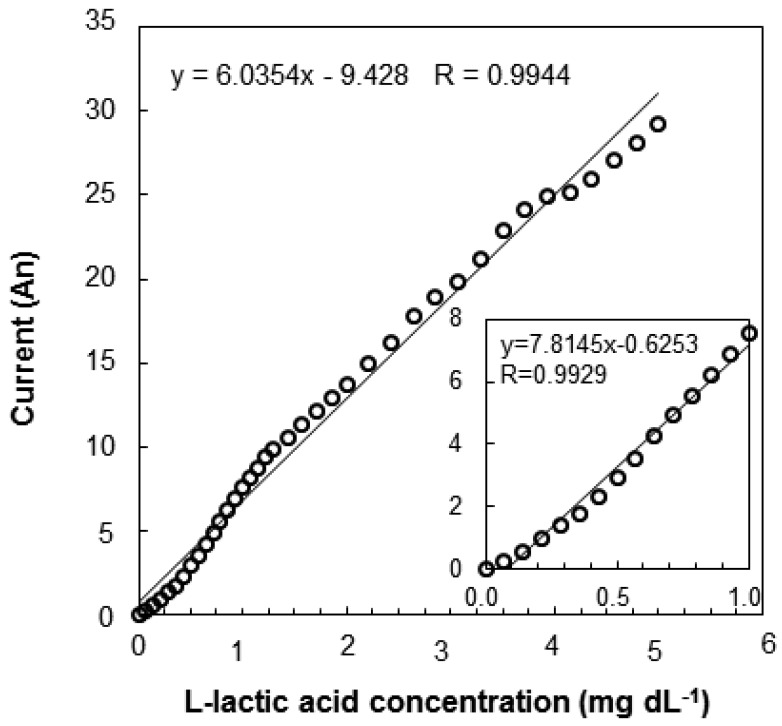
Calibration curves of the biosensor in EISF samples.

Fish are poikilothermic, and their temperature depends on the temperature of the rearing water. The water temperature used for rearing Nile tilapia ranges from 24 to 28 °C. Thus, the sensor induces sufficient output current under typical EISF temperature conditions and can be applied to *in vivo* measurements. The reproducibility of sensor measurement is shown in Table 1. The reproducibility of the measurement was demonstrated by immersing a single sensor in 1,000 mg·dL^−1^ standard solution (30 °C). Each of the three enzyme sensors was tested 25 times. The percent relative standard deviation (RSD%) was used to evaluate the reproducibility to account for lot-dependent variability in the sensor output current. The RSD% was 7.86 ± 0.53% for the absolute response values of the three sensors. This RSD% value indicates that the sensor cannot be used to determine l-lactic acid concentrations with satisfactory precision. The differences between lots is probably due to the fact that the sensors are handmade one at a time. Commercializing the manufacturing process of the sensor should dramatically decrease the inter-lot variability. Inter-lot differences can be overcome to obtain an accurate measurement by making a calibration curve for each sensor. Figure 7 shows an evaluation of the needle-type L-lactic acid sensor in EISF samples (L-lactic acid concentration in EISF; 1.704 mg·dL^−1^). A calibration curve of the biosensor measuring EISF L-lactic acid levels is also shown in Figure 7. The sensor had a good linearity in the range from 1.7 to 5.0 mg·dL^−1^. Linear regression analysis revealed a very strong correlation coefficient of 0.9944. Thus, the sensor can be used to measure L-lactic acid in EISF.

### 3.3. Short Duration Continuous l-Lactic Acid Monitoring Using a Wireless Enzyme Sensor System under Free-Swimming Conditions

**Figure 8.**
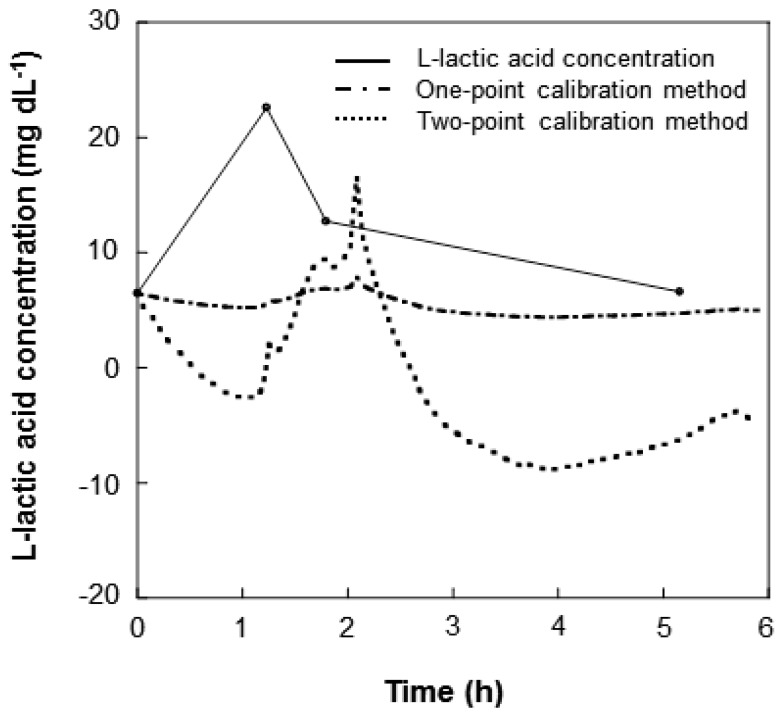
Relationship between l-lactic acid concentration in EISF and the calibrated sensor response. The l-lactic acid concentrations are plotted as circles, the calibrated response using the one-point calibration method is plotted as a broken line, and the calibrated response using the two-point calibration method is plotted as a solid line.

We performed continuous l-lactic acid monitoring in Nile tilapia for 6 h using a wireless sensor system. The accuracies of the one-point and two-point calibration methods were compared (Figure 8). The one-point calibration method could accurately calculate the EISF lactic acid concentration based on the output current value and thereby provide an estimation of the blood l-lactic acid concentration. It is not possible to state, however, whether a real time correlation exists between the EISF and blood lactic acid concentrations due to the time-lag in the change of l-lactic acid concentration between the blood and the EISF. Approximately 1 h was required to register an increase in the EISF l-lactic acid levels following an increase in the blood l-lactic acid levels. Despite the time-lag, the l-lactic acid concentration could be accurately determined.

Using the two-point calibration method the l-lactic acid concentration in the blood is converted from the output current value of the sensor and the actual l-lactic acid concentration in blood is shown. The two-point calibration method was adopted using the first measurement (*I*_1_; 2.16 nA, l-lactic acid; 6.47 mg dL^−1^) and a second measurement obtained 100 min later (*I*_1_; 2.45 nA, l-lactic acid; 12.72 mg dL^−1^). The converted value is negative, however, because the rate of increase of the l-lactic acid concentration in EISF is lower than that of the l-lactic acid concentration in the blood. Therefore, the one-point calibration method was simple and more effective and should be used to measure l-lactic acid concentrations in fish.

### 3.4. l-Lactic Acid Long Time Monitoring Using the Wireless Enzyme Sensor System under Free-Swimming Conditions

**Figure 9.**
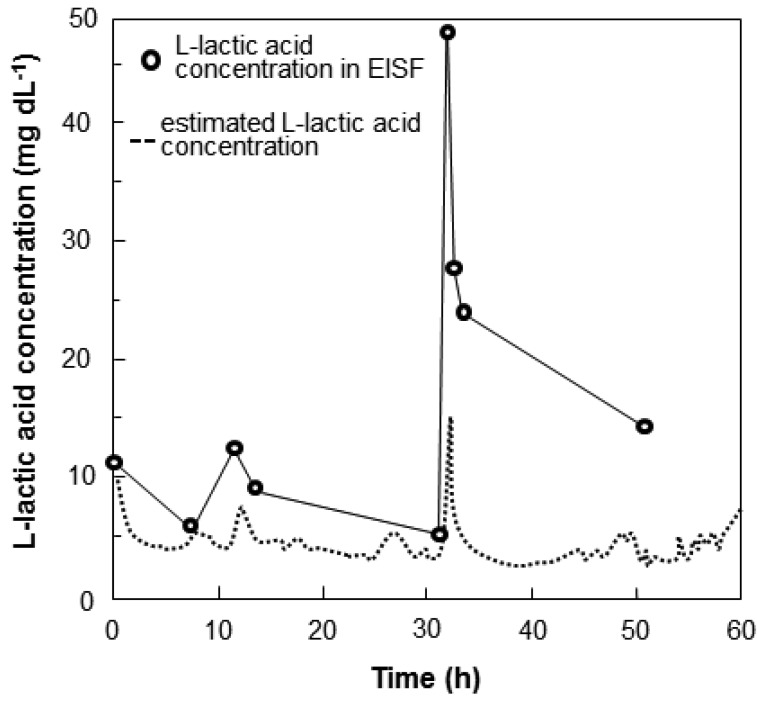
Change in the estimated l-lactic acid concentration based on the wireless monitoring system. The l-lactic acid concentrations in the EISF at 0 min, plotted as black circles, were used for the one-point calibration method.

Nile tilapia were anesthetized with 400 ppm 2-phenoxyethanol, and then the wireless sensor system was attached to the fish. The fish were transferred to a 50-L fish water tank containing no anesthetic. The time course of the output current of the sensor and sensor-calibrated l-lactic acid levels are shown in Figure 9. Initially, the l-lactic acid levels were slightly high, which may have been caused by stress induced by fixation of the sensor or by the sensor itself. The lactic acid levels decreased gradually and stabilized after 5 to 6 h. When the fish were stressed by adding 2-phenoxyethanol to the water at 8 h and 22 h after beginning recording, the l-lactic acid concentration in the blood and in the EISF of fish increased. Although the blood lactic acid levels increased rapidly to 50 mg·dL^−1^, the lactic acid in EISF increased only up to 20 mg·dL^−1^. The maximum measurement limit of the sensor was approximately 30 mg·dL^−1^ (Figure 7). Blood lactic acid levels of 50 mg·dL^−1^ are considerably abnormal. The sensor functions sufficiently, because the lactic acid measurements in the EISF had the same trend as those in the blood. Our proposed sensor provides rapid and convenient real-time wireless monitoring of l-lactic levels in fish for over 60 h. The results of the wireless monitoring show the potential of the sensor as a ubiquitous tool for use in the aquaculture industry.

## 4. Conclusions

In the present study, we developed a needle-type enzyme biosensor system to continuously monitor l-lactic acid concentrations in fish. We used EISF as an alternative to blood and investigated the relationship between l-lactic acid concentration in the blood and EISF. The l-lactic acid concentration in EISF correlated with that in blood. We prepared a needle-type enzyme sensor for measuring l-lactic acid in fish. The sensor was small (0.4 mm width × 20 mm length) and was inserted in the vicinity of the fish eyeball. We evaluated two calibration methods for measuring l-lactic acid in fish. The one-point calibration method was sufficiently accurate for this measurement system. Using the one-point calibration method, we successfully monitored blood l-lactic acid levels of the fish for 60 h. The purpose of this study was to monitor stress in fish by monitoring the l-lactic acid concentration in blood. In monitoring the l-lactic acid concentrations in fish, we confirmed that blood l-lactic acid levels increased in the blood when an external stressor was added to their environment. We have been measuring blood glucose and cholesterol levels as markers of stress in fish. By combing various indices, it becomes possible to more accurately assess the health condition of free-swimming fish.

## Figures and Tables

**Table 1.** Reproducibility of l-lactic acid measurements using the l-lactic acid biosensor (n = 25). The measurement was performed using 5 mg·dL^−1^ l-lactic acid in PBS, pH 8.0, 30 °C under a +650 mV potential (*vs.* Ag/AgCl).

**Electrode No.**	**Relative standard deviation (RSD %)**
1	7.37
2	7.78
3	8.43
Mean ± SD	7.86 ± 0.53
